# A pseudo-phased genome assembly for *Hemileia vastatrix* reveals an isolate-specific chromosomal haploid trisomy

**DOI:** 10.1093/g3journal/jkag214

**Published:** 2026-08-01

**Authors:** Peri A Tobias, Richard J Edwards, Jamie Botting, Giullia di Lorenzo, Vera Inácio, Inês Diniz, Maria do Céu Silva, Leonor Guerra-Guimarães, Vitor Várzea, Robert F Park, Dora Batista

**Affiliations:** School of Life and Environmental Sciences, The University of Sydney, Camperdown, NSW 2006, Australia; Ocean Genomes Laboratory, Minderoo OceanOmics Centre at University of Western Australia Oceans Institute, the University of Western Australia, Perth, WA 6009, Australia; Ocean Genomes Laboratory, Minderoo OceanOmics Centre at University of Western Australia Oceans Institute, the University of Western Australia, Perth, WA 6009, Australia; Centro de Investigação das Ferrugens do Cafeeiro (CIFC), Linking Landscape, Environment, Agriculture and Food (LEAF), Associate Laboratory TERRA, Instituto Superior de Agronomia, Universidade de Lisboa, Lisboa 1349-017, Portugal; Centro de Investigação das Ferrugens do Cafeeiro (CIFC), Linking Landscape, Environment, Agriculture and Food (LEAF), Associate Laboratory TERRA, Instituto Superior de Agronomia, Universidade de Lisboa, Lisboa 1349-017, Portugal; Centro de Investigação das Ferrugens do Cafeeiro (CIFC), Linking Landscape, Environment, Agriculture and Food (LEAF), Associate Laboratory TERRA, Instituto Superior de Agronomia, Universidade de Lisboa, Lisboa 1349-017, Portugal; Centro de Investigação das Ferrugens do Cafeeiro (CIFC), Linking Landscape, Environment, Agriculture and Food (LEAF), Associate Laboratory TERRA, Instituto Superior de Agronomia, Universidade de Lisboa, Lisboa 1349-017, Portugal; Centro de Investigação das Ferrugens do Cafeeiro (CIFC), Linking Landscape, Environment, Agriculture and Food (LEAF), Associate Laboratory TERRA, Instituto Superior de Agronomia, Universidade de Lisboa, Lisboa 1349-017, Portugal; Centro de Investigação das Ferrugens do Cafeeiro (CIFC), Linking Landscape, Environment, Agriculture and Food (LEAF), Associate Laboratory TERRA, Instituto Superior de Agronomia, Universidade de Lisboa, Lisboa 1349-017, Portugal; School of Life and Environmental Sciences, Plant Breeding Institute, The University of Sydney, Narellan NSW 2567, Australia; Centro de Investigação das Ferrugens do Cafeeiro (CIFC), Linking Landscape, Environment, Agriculture and Food (LEAF), Associate Laboratory TERRA, Instituto Superior de Agronomia, Universidade de Lisboa, Lisboa 1349-017, Portugal

**Keywords:** coffee leaf rust, Hv178a, *Coffea arabica*, plant pathogen

## Abstract

Recurrent epidemics of coffee leaf rust, caused by the fungal pathogen *Hemileia vastatrix,* have constrained production of Arabica coffee for over 150 years. Here, we present a pseudo-phased, chromosome-level genome resource for *H. vastatrix,* isolate Hv178a, to guide research into disease management. The Hv178a genome assembly is 665 and 638 Mbp for haplotype A and B, respectively, localized to 18 chromosomes. We determined that the genomes are highly repetitive at ∼94%, with a guanine-cytosine (GC) content of 33.5%. We present the full annotation of 13,760 and 17,998 protein-coding genes, and we predicted 452 and 496 effectors in haplotype A and B, respectively. Depth-based comparisons with 11 additional *H. vastatrix* isolates revealed increased chromosome 17 (chr17) copy number in Hv178a. qPCR validation supports a chr17 trisomy in Hv178a absent from the ancestral lineage and potentially explaining the observed change in virulence.

## Teaser

Tobias *et al*. present the first chromosome-level and pseudo-phased genome assembly for the coffee leaf rust pathogen, *Hemileia vastatrix*, to guide research into disease management. Using isolate Hv178a, the authors confirm the conservation of 18 haploid chromosomes, a feature common across distantly related rust genomes. The genome has a very high repeat (94%) and low GC (33.5%) content in line with other expanded rust genomes. Comparative depth-based analyses against 11 isolates, validated via qPCR, revealed a unique chromosome 17 trisomy in Hv178a. This structural variation is absent in the ancestral lineage and potentially explains a changed virulence profile for Hv178a.

## Introduction

The specialized fungal pathogen *Hemileia vastatrix* Berk. & Broome (phylum Basidiomycota, class Pucciniomycetes, order Pucciniales), causing the devastating disease coffee leaf rust (CLR), has been the major constraint to Arabica coffee (*Coffea arabica*) production for more than 1 and a half centuries (McCook and Vandermeer 2015; Cabral et al. 2016; Talhinhas et al. 2017). First recorded by an English explorer in 1861 near Lake Victoria (East Africa) on wild *Coffea* species, CLR wiped out Arabica coffee cultivation from Ceylon (Sri Lanka) only 5 yr later, with devastating social and economic consequences (Morris 1880). Since this historical first outbreak, the disease gained a worldwide distribution, spreading progressively through coffee production areas of Asia and Africa and, finally, crossing the Atlantic Ocean where it expanded across Latin America (McCook 2006). With the more recent detection of CLR in Hawaii in 2020 (Keith et al. 2021), the disease became endemic in all coffee-producing regions of the world. Although the development and cultivation of rust-resistant varieties have contributed to CLR control, high adaptation of the fungus has led to recurrent and severe epidemics resulting in heavy yield and revenue losses. Reports estimate production losses more than $1 billion annually worldwide (Kahn 2019).

Due to the ability of the pathogen to respond rapidly to selection pressure, more than 55 physiological races have been identified to date (Silva et al. 2022). The rapid evolution of *H. vastatrix* to overcome resistance in coffee cultivars is more puzzling considering that the pathogen exists almost exclusively in the asexual stage of its life cycle and that the dikaryotic urediniospores are the only known functional propagules (Talhinhas et al. 2017; Koutouleas et al. 2019; Silva et al. 2022). The dikaryotic stage, whereby 2 separate haploid nuclei are present within the urediniospore cytoplasm, provides a unique opportunity to investigate the potential independent genome evolution of each nucleus during asexual reproduction in the rust fungi. Recent haplotype-resolved assemblies have revealed that the 2 nuclei can exhibit substantial genomic divergence and internuclei variation, including chromosome reassortment (Luo et al. 2025), mating gene arrangement and strategy (Ferrarezi et al. 2022), and somatic nuclear exchanges (Sperschneider et al. 2023). Deconstructing the evolutionary complexity between *H. vastatrix* and its only known host is crucial to developing options for disease control. Such goals rely on the availability of a comprehensive and high-quality genome reference assembly. The isolate used for the current research is a mutant virulent strain obtained in 1960 from the greenhouse complex of Centro de Investigação das Ferrugens do Cafeeiro (CIFC), named CIFC Hv178a (Fig. 1).

**Fig. 1.**
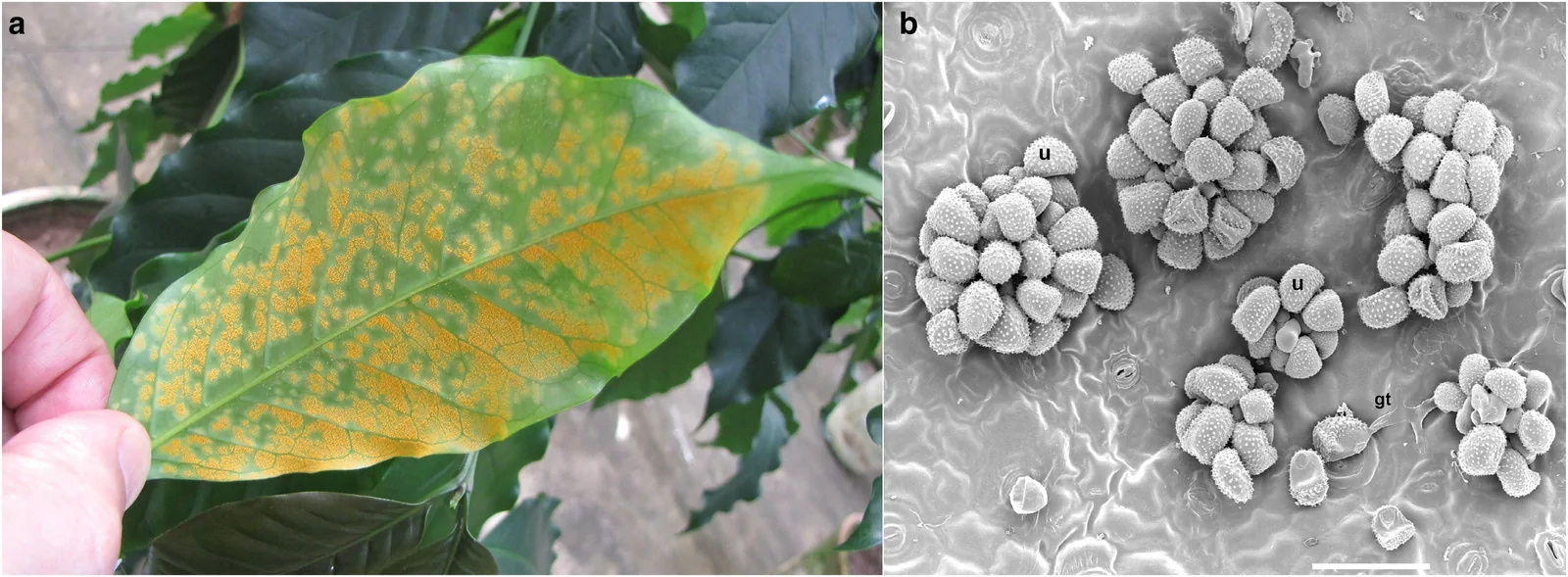
a) *Coffea arabica* leaf with symptoms of coffee leaf rust disease caused by the fungal pathogen *Hemileia vastatrix* at the Centro de Investigação das Ferrugens do Cafeeiro (CIFC) greenhouse complex, Universidade de Lisboa, Portugal in 2022. b) Surface view of superstomatal uredosporic sori of *H. vastatrix*. Note the reniform urediniospores (U), and an early stage of germination (gt = germ tube). Scanning electron microscopy (Bar = 50 µm) (HG Azinheira and MC Silva, unpublished.).

Here, using additional chromosome confirmation capture sequence data, we manually curated to improve on a previous haploid genome assembly (Tobias et al. 2022) and report a pseudophased and chromosome-level genome for *H. vastatrix* strain CIFC Hv178a, henceforth Hv178a. We confirm the conservation of 18 haploid chromosomes within *H. vastatrix*, in line with numbers determined for distantly related rust fungi (Schwessinger et al. 2022; Luo et al. 2024). We compared the assembled genome to data from 11 other isolates, including the ancestral Hv178, and determined intriguing chromosome copy number variation indicating trisomy for chromosome 17 (chr17), validated with qPCR, potentially explaining the observed change in virulence. The genome provides a high-quality resource for advancing knowledge on the complex coffee rust pathogen as well as identifying a novel chromosomal anomaly for this isolate.

## Methods

### Fungal material

*H. vastatrix* isolate Hv178a (type specimen of race XIV: virulence profile v_2,3,4,5_), maintained at the spore collection of CIFC, Instituto Superior de Agronomia, Universidade de Lisboa, was used in this work. This isolate is a mutant strain obtained in 1960 at CIFC’s greenhouse complex derived from isolate CIFC Hv178 (race VIII: virulence profile v_2_,_3,5_), collected in India (Balehonnur, Coffee Research Station) in 1958 from *C. arabica* S.288-23. Within the scope of experimental selection assays for increased virulence, the repeated inoculation of Hv178 onto a resistant Arabica coffee plant led to the emergence of a new isolate, Hv178a, bearing the addition of v4 to its virulence spectrum (Bettencourt 1981). Hv178a was multiplied on its differential host plant (*C. arabica* accession CIFC H147/1, carrying the resistance factors S_H_2,3,4,5), as previously described (d’ Oliveira 1954). Single urediniospore pustules were collected, frozen in liquid nitrogen, and stored at −80 ˚C until further processing.

### DNA extraction and sequencing

High molecular weight (HMW) genomic DNA was extracted using a cetyltrimethylammonium bromide (CTAB)-based modified protocol (Schwessinger and Rathjen 2017). DNA samples were further purified using Pacific Biosciences (PacBio) SampleNet—Shared Protocol (https://www.pacb.com/wp-content/uploads/2015/09/SharedProtocol-Extracting-DNA-usinig-Phenol-Chloroform.pdf). DNA concentration and purity were measured using a Multiskan SkyHigh Microplate Spectrophotometer (Thermo Fisher Scientific) and a Qubit 4 Fluorometer (Thermo Fisher Scientific), and its quality was checked on an agarose gel. Ten micrograms of high-quality HMW DNA were sent to CD Genomics (New York, United States) for SMRTbell size select long read library preparation and PacBio Sequel II sequencing. The same sample of HMW DNA was used for library construction with TruSeq DNA PCR-Free kit, following manufacturer's recommendations, and resequencing with paired-end 151 bp reads at CD Genomics on an Illumina NovaSeq platform.

### Cross-linking of spores and chromatin conformation capture sequencing

To improve the scaffolding of our genome assembly, we obtained chromatin conformation sequence data (HiC). About 400 mg of Hv178a urediniospores were crosslinked in a 1% formaldehyde solution for 20 min at room temperature. Crosslinking was quenched by adding glycine to a final concentration of 125 mM. The fixed tissue was then ground in liquid nitrogen and the stored frozen powder sent to Phase Genomics (Seattle, WA) for nuclei isolation, HiC library preparation and sequencing. Data were generated using a Phase Genomics (Seattle, WA) Proximo Hi-C 4.0 Kit, which is a commercially available version of the HiC protocol (Lieberman-Aiden et al. 2009) using the DPNII restriction enzyme. Sequencing was performed on an Illumina HiSeq 4000, generating a total of 175,294,501 paired-end (PE) 150 read pairs (total reads 350,589,002).

### Draft genome assembly

We received raw PacBio Sequel (Menlo Park, California, United States) SMRT continuous long reads from CD Genomics (New York, United States). Prior to correction, we had 7,539,679 reads (166 gigabases), with a median read length of 17,992. This provided approximately 200 times coverage on an expected genome size of 800 Mb, based on flow cytometry (Tavares et al. 2014). An earlier version of the Hv178a isolate genome assembly using this Sequel data was made public (Tobias et al. 2022). The genome indicated a potential *associated fungal genome* (AFG) or contaminant, and this caused problems when attempting to phase the diploid assemblies due to high levels of duplicated gene models. Additionally, our earlier version was scaffolded with limited HiC data, only 31,666,891 PE reads. We therefore decided to reassemble the genome, curate, and scaffold with adequate HiC read data coverage. We used Canu (v2.1.1) (Koren et al. 2016) with the following recommended parameters for diploid genome assembly; genomeSize = 800 m, corOutCoverage = 100, corMaxEvidenceErate = 0.15, correctedErrorRate = 0.040, batOptions = “-dg 3 -db 3 -dr 1 -ca 500 -cp 50”. These options produced an assembly of 1.71 Gb with 6,088 contigs, an N50 of 74,5640 and L50 of 438. Genome outputs were tested for contiguity with Quast version 5.0.2 (Gurevich et al. 2013) and conserved single-copy gene completeness with Benchmarking Universal Single-Copy Ortholog (BUSCO) (v3.0) (Simão et al. 2015) with the Basidiomycota_odb9 database. We used this output going forward.

### Genome deduplication

We ran Purge Haplotigs (v1.0) (Roach et al. 2018) to deduplicate the 1.7 Gb genome using these counts per read depth; -l 32 -m 115 -h 185 after mapping with Minimap2 (v2.3) (Li, 2018). We used these purge parameters for the cutoffs; -I 250 M -a 75 and produced 2 genome fasta outputs with the following BUSCO statistics; C:93.1%[S:57.3%, D:35.8%], F:2.4%, M:4.5% and C:71.0%[S:49.7%, D:21.3%], F:4.1%, M:24.9% for the primary (575 M) and haplotig (785 M) assemblies. The BUSCO statistics indicated difficulties in separating the haplotypes and the continued presence of the contaminant AFG sequence, with large numbers of duplicated conserved genes in each assembly.

### Assembly decontamination

The goals of the initial assembly curation were to (i) remove the AFG sequences and any other contamination, and (ii) purge excess haplotigs. Due to the complications arising from the Purge Haplotigs run, the original Canu primary (464 contigs, 596.4 Mbp) and alternative (2,261 contigs, 814.7 Mbp) assemblies were used as the basis for curation. Sequencing reads were mapped onto the earlier Hv178a isoate genome assembly (Tobias et al. 2022), using Minimap2 (v2.2) (Li, 2018) for PacBio and Burrows-Wheeler Aligner Maximal Exact Matches (BWA-MEM) (v0.7.17) (Li, 2013) for Illumina reads. Mapped reads were partitioned using Samtools (v1.15) (Li et al. 2009) into (i) AFG reads, (ii) mtDNA reads, (iii) nuclear reads. PacBio nuclear reads were mapped independently onto the primary and alternative Canu assemblies using Minimap2 (v2.2) (Li, 2018). Any contigs with below 3X mean coverage were removed from the assembly as likely contamination (eg AFG) or mtDNA. In addition, Tiara (v1.0) (Karlicki et al. 2022) identified 1 mitochondrial and 2 bacterial contigs, which were removed from the assembly. Next, Diploidocus (v1.5.0) (Chen et al. 2022) was run on each haplotype using the filtered nuclear PacBio reads for depth calculations and the filtered nuclear Illumina reads for k-mer analysis. Genome completeness was estimated using BUSCO (v5.1.2) (Manni et al. 2021) with the basidiomycota_odb10 database. Following removal of the low-coverage contigs, the haplotypes were 64.4% (pri) and 68.8% (alt) complete, with 15.9% and 23.6% duplication. However, the combined diploid assembly was 85.5% complete and 80.6% duplicated, suggesting that many BUSCO-containing contigs had both haplotigs assigned to the same haploid assembly. This was exacerbated by Diploidocus, which overpurged because of these false duplications, reducing the diploid BUSCO completeness to 82.9% complete and 50.1% “duplicated”.

### Assembly phase correction and curation

The Diploidocus cleanup erroneously purged contigs that were misassigned to haplotypes rather than falsely duplicated in the overall assembly. To compensate for this, the quarantined contigs from each haploid assembly were added back to the other assembly (eg contigs purged from the pri assembly were added to the alt assembly and vice versa). Haplotypes were renamed as hap1 (83.35% Complete, 9.4% Duplicated) and hap2 (76.1% Complete, 5.4% Duplicated), and each haploid assembly subjected to a second round of Diploidocus tidying. This was more successful but still suffered from an overall loss of diploid BUSCO Complete and Duplicated genes. Contigs were therefore manually filtered using Diploidocus sequence statistics and identifying contigs with common BUSCO genes. Where a quarantined contig contained a BUSCO gene that was found twice in 1 haplotype but not in the other, it was moved to the other haplotype. Excess copies (eg once both haplotypes contained the genes) were marked for removal as usual. Contigs were dealt with in size order, largest first, so the smaller contig was always marked for removal. Next, underquarantined contigs that contained within-haplotype Duplicated BUSCO genes but had not been marked for removal were processed in a similar fashion, moving 1 copy when the other haplotype lacked the corresponding BUSCO genes or marking it for removal when it did. The exception was contigs that also contained single-copy BUSCO genes, or Duplicated BUSCO genes that were rated as True Duplicates based on DepthKopy (Chen et al. 2022) read depths. These contigs were retained in the original haplotype. After phase correction, the 2 haploid assemblies were 83.6 and 78.3% Complete, with 1.9% Duplicated BUSCOs in each case. The combined diploid was 85.3% Complete and 76.6% Duplicated, indicating that most of the assembly was now partitioned correctly into 2 haplotypes. It should be noted, however, that some loss and misassignment of contigs is likely to remain. These haploid assemblies were then scaffolded to chromosome-level using the HiC data.

### Chromosome-scale scaffolding with HiC reads

Several strategies were employed to scaffold and phase the genomes using HiC data. Initial attempts with the concatenated genomes were not successful and tended to collapse the genome and decrease contiguity and completeness. We consider that this is due to the error-prone nature of the early-generation PacBio Sequel data leading to assembly issues, as has been previously shown for such data with large and repeat-rich dikaryotic rust fungi (Tobias et al. 2021). We therefore proceeded to independently scaffold the haploid assemblies using the Aiden Lab pipelines (https://github.com/aidenlab). Firstly, the files were run through the Juicer pipeline (v1.6) (Durand et al. 2016) with default parameters. The final output from Juicer was used with the 3D-DNA pipeline (v180922) (Dudchenko et al. 2017) with the following parameters: “-r 1-m haploid –build-gapped-map –sort-output”. The first-round HiC and assembly files were manually curated locally within the Juicebox visualization software (v1.11.08 for Windows) (Durand et al. 2016), and the revised assembly file was resubmitted to the 3D-DNA post-review pipeline with the following parameters: “–build-gapped-map –sort-output” for final assembly and fasta files.

### Post-scaffolding assembly curation

Following Hi-C scaffolding, chromosome ends were assessed with Telociraptor (v0.9.0) (https://github.com/slimsuite/telociraptor) to correct for telomere-masking inversions or to remove contigs scaffolded onto telomeres. Five chromosome ends in hap1 and 7 in hap2 were corrected to reveal telomeres. ChromSyn (Edwards et al. 2022) analysis revealed 2 hap2 chromosomes with arms mis-scaffolded end-to-end, which were also corrected with Telociraptor. ChromSyn and Telociraptor were also used to pair and rename hap1 and hap2 chromosomes. Each haploid assembly was then polished with Hypo (Kundu et al. 2019) using the partitioned PacBio and Illumina reads mapped onto the diploid assembly. Finally, Diploidocus was run using reads mapped onto the diploid assembly to identify and remove any haplotype-specific contigs that had been falsely duplicated, or unplaced “debris” contigs that were redundant following HyPo gap-filling.

### Genome quality assessment

We estimated the functional completeness of the genome using BUSCO (v5.1.2) with gene predictions (Levy Karin et al. 2020; Manni et al. 2021) in genome mode and the database basidiomycota_odb10. Subsequently, we ran both genome and protein modes with database BUSCO (v6) basidiomycota_odb12 (Tegenfeldt et al. 2025). Basic genome statistics were determined with Quast (v5.0.2) (Gurevich et al. 2013). K-mer-based genome sequence completeness and accuracy were estimated with Merqury (v1.3) (Rhie et al. 2020).

### Mitochondrial genome assembly

We previously assembled and made public the complete circularized mitochondrial sequence (Tobias et al. 2022). We included the assembled Hv178a_MTDNA *H. vastatrix* isolate = Hv178a as a final contig within the principal pseudo-haplotype lodged at the National Center for Biotechnology Information (NCBI): JAMZDC000000000.

### Read depth genome size prediction and copy number analysis

Genome size predictions were made with DepthSizer (v1.6.3) (Chen et al. 2022), using partitioned PacBio reads (see below) mapped with Minimap (v2.4) (Li, 2018) and BUSCO (v5.1.2) (Manni et al. 2021) gene predictions (see above). Read depth statistics and predicted copy numbers were generated using DepthKopy (v1.0.3) (Chen et al. 2022).

### Repeat family annotation

Transposable elements and simple sequence repeats were annotated with RepeatModeler (v2.0.1) (Smit and Hubley 2019) and RepeatMasker (v4.0.6) (Smit et al. 2019). Initial repeat libraries were constructed for the complete haplotype A genome and then used to predict repeats within both haplotype A and B with RepeatMasker using the following parameters: -pa 24 -gff -xsmall –lib. The softmasked output genome was used to map RNAseq data and for downstream annotation. We provide the Hv178a repeat data gff files here: https://osf.io/wtn4 h/files/osfstorage.

### Protein annotation with RNA data from a closely related isolate

We used RNAseq data from the ancestral isolate, Hv178 (PRJNA1483988), as evidence for annotating predicted coding sequences. First, we combined the Illumina paired-end RNAseq data derived from 3 replicates of the isolate Hv178 (v_2,3,5_) during compatible interaction with the respective differential host plant (*C. arabica* accession CIFC 34/13, S_H_2,3,5) at 3 key stages of infection, namely at 1, 4, and 10 d postinoculation. The raw Illumina data was first trimmed of adaptors and low-quality reads using default settings with fastp (v0.19.6). We then mapped the data to the masked genomes of haplotype A and B independently using hisat2 (v2.1.0) with “–no-unal” and all other defaults. The resulting bam files were incorporated as evidence for annotation with Funannotate (v1.8.15) (Palmer and Stajich 2023) with the predict function and using the BUSCO dikarya_odb9 database (Simão et al. 2015) with diamond (v2.1.6) (Buchfink et al. 2021). The predicted amino acid (aa) fasta files for each haplotype were then put through Interproscan (v5.52-86.0) (Jones et al. 2014) to determine putative functions with default database settings. The same aa fasta files were screened with SignalP (v4.1f) (Nielsen 2017) and the subsequent 797 haplotype A and 891 haplotype B proteins with EffectorP (v3) (Sperschneider and Dodds 2022) to identify predicted effectors.

### Haplotype ortholog investigation

Predicted protein fasta files were uploaded to the Orthovenn3 (Sun et al. 2023) webserver to investigate orthology and collinearity of haplotype chromosome-level scaffolds. The 2 haplotypes share 7,706 ortholog clusters. We determined 8,061 ortholog clusters, including 289 unique orthogroups (OG) in haplotype A and 4,099 singleton proteins that do not fall into any cluster. We determined 8,368 ortholog clusters, including 662 unique OG in haplotype B and 5,383 singleton proteins that do not fall into any cluster. We then checked the list of singletons from each haplotype for evidence of transposable element (TE) functional annotations using the TSV output from interproscan withword matches “transposase, reverse transcriptase, integrase, retrotransposon, mariner, muDR, gypsy, copia, LINE-1”. We compared the ratio of unique genes for TE-type sequences to all unique functional predictions within the singletons and within the full InterProScan output for TE enrichment from each haplotype. We found no statistical significance of TE enrichment using a Fisher's Exact Test in either haplotype. Comparative gene copy number analysis between Hv178a and 11 other isolates.

We investigated copy number (CN) of conserved single-copy genes using Illumina data from Hv178a and 11 other *H. vastatrix* isolates: Hv23, Hv70, Hv71, Hv178, Hv264a, Hv741, Hv995, Hv999, Hv1427, Hv3305 (PRJNA1484073), and Hv race I (Ángel C. et al. 2023). For each strain, paired-end read data were mapped onto Hv178a haplotype A using BWA-Mem (Li, 2013). The CN for each gene in each strain was estimated using DepthKopy (Chen et al. 2022), based on empirical single-copy read depth estimates derived from single-copy BUSCO (v5.4.4, odb10) predicted genes. The copy number ratio (CNR) for each gene was then calculated as the ratio of the CN for Hv178a vs the mean CN for all other strains. The data was subject to a log2 transformation, where zero represents no CN difference, and a value of +/− 1 represents a 2-fold difference. To visualize clusters of genes with a difference in fold change, the log2 change was plotted along the relevant chromosome physical position using ggplot in R (v4.4.1) (The R Core Team 2026). Plots were created for each chromosome using the gene's location with the positive fold change above 0 and negative fold change below 0. The mean, 1 standard deviation, 2 standard deviations, and 3 standard deviations were calculated using the individual chromosome gene copy numbers. Additionally, a protein domain analysis was done to observe any missed changes in protein families or single genes that have generated a false high copy number. This was achieved by collapsing the integrated data from DepthKopy, Funnannotate, and Pfam domain annotations onto each Pfam protein domain family. The mean CN was then used to calculate any fold change between the mean domain CN of other isolates compared to Hv178a.

### Copy number estimation by quantitative real-time PCR (qPCR)

#### Primer design, insert amplification and cloning

Validation of genomic CN prediction in chr17 was tested with qPCR analysis in isolate Hv178a compared with Hv178. Three gene targets were used: a predicted effector located in chr17A (Hv178aA_012933-T1), a conserved single copy gene located in chr17A (putative fluoride ion transporter CrcB—locus 13767at5204) and a conserved single copy gene located in chr 6A (Actin-related protein 2-Mar complex subunit 5—locus 82138at5204), denoted as E33, B17.1 and BC6, respectively. BUSCO conserved gene loci are available as Supplementary Data (https://osf.io/wtn4h/files/osfstorage). Four biological replicates of DNA extracted from isolates Hv178a and Hv178, as previously described (Silva et al. 2018), were used. Primers were designed using Primer Select (DNASTAR Lasergene, v 11.1.0.54). Amplification of the E33, B17.1 and BC6 gene fragments to use as cloning inserts was performed using DNA template from isolate Hv178a under the following conditions: 20 μL reactions containing 4 μL of GoTaq Flexi buffer (1X, Promega, Madison, Wisconsin, United States), 2 μL of MgCl_2_ (25 mM, Promega, Madison, Wisconsin, United States), 0.5 μL of dNTPs mix (10 μM, Thermo Fisher Scientific, Waltham, Massachusetts, United States), 0.5 μL of BSA (10 mg/mL, GeneOn, Germany), 1 μL of each forward and reverse primers (10 mM, StabVida, Caparica, Portugal), 0.2 μL of GoTaq Flexi DNA Polymerase (5 u/μL, Promega, Madison, Wisconsin, United States), 3 μL of DNA template (15 ng/μL) and water to the final volume. A negative control with no template was included. PCR was carried out with an initial denaturation step at 94 °C for 3 min, followed by 35 cycles of denaturation at 94 °C for 1 min, annealing at the respective temperature for each gene (Table 1) for 1 min, and extension at 72 °C for 1 min, and 1 final step of extension at 72 °C for 7 min. PCR products were purified using the Wizard SV Gel and PCR Clean-Up System kit (Promega, Madison, Wisconsin, United States), quantified in a Qubit 4 Fluorometer (ThermoFisher Scientific, Waltham, Massachusetts, United States) and cloned into the vector and *E. coli* JM109 competent cells using the pGEM-T Easy Vector System II Systems Kit according to the manufacturer's instructions (Promega, Madison, Wisconsin, United States), in a 3:1 (insert:vector) molar ratio. Positive colonies were confirmed by colony PCR using the universal primers SP6 and T7 and the specific primers for the inserts (E33, B17.1, BC6, Table 1). The cloned plasmids were isolated from pelleted cell cultures using Wizard *Plus* SV Minipreps DNA Purification kit (Promega, Madison, Wisconsin, United States) and sequenced (Stabvida, Caparica, Portugal) to verify the presence and the correct insertion of the fragments in the vectors.

**Table 1.** Primer sequences for qPCR analysis of *Hemileia vastatrix* target genes.

Locus	Gene	Description	Primer sequence (5’-3’)	Amplicon length (bp)	Ta (°C)	PCR efficiency (%)
12,933	E33	Predicted effector	Fw: CGTCTTAGCGGCAGGATTAC Rv: AGGGGGGATTTTGTTGATTGG	79	60	93
13,767	B17.1	Putative fluoride ion transporter CrcB	Fw: CCAAGATGATAACGAAGTAATG Rv: AGAAATGAAGGAGGAAAGAGTA	197	55	91
82,138	BC6	Actin-related protein 2	Fw: AAACGCGCATAATACAACCTG Rv: TTTCTCTTTACCCCCACTTCT	138	50	92

#### Construction of standard curves for determination of gene copy numbers

Plasmid DNA samples extracted from cell cultures cloned with E33, B17.1, and BC6 gene fragments were quantified using Qubit 1X dsDNA BR Assay Kit (Thermo Fisher Scientific, Waltham, Massachusetts, United States) and first diluted serially to 1 and 0.01 ng/µL. The corresponding copy number of plasmid DNA per microliter was calculated using the following equation (Whelan et al. 2003):


$$DNA\;copy\;=\;\frac{6.02\times 10^{23}(copy/mol)\times DNA\;amount\;(g)\;}{DNA\;length(bp)\times 660\;(g/mol/bp)}$$

From a dilution of 100 copies/µL (0.01 ng/µL), subsequent dilutions were prepared for each target gene, corresponding to 75, 50, 25, 20, 15, 10, 5, 3, 2, 1.5, and 1 copy/µL. Dilutions from 100 to 1 plasmid copy/µL were run on a CFX Connect Real-Time PCR Detection System (Bio-Rad, Hercules, California, United States) with the following conditions: 20 µL of reaction mixture prepared with 10 µL of SsoFast EvaGreen Supermix (Bio-Rad, Hercules, California, United States), 0.8 µL of each forward and reverse primers (0.01 mM), 1 µL of DNA template and water to the final volume. All qPCR experiments were carried out with the following thermal cycling protocol: an initial denaturation step at 95 °C for 3 min, followed by 41 cycles of denaturation at 95 °C for 15 s, annealing and extension at Ta (Table 1) for 30 s. In each set of reactions, at least 3 replicates per copy dilution were run, in addition to a non-template control. A standard curve for each gene was generated by plotting quantification cycle (Cq) average values (with standard deviation < 0.5) of each dilution step against the corresponding log10 transformed number of gene copies in the standard. Amplification efficiencies (E) were determined using LinRegPCR software (v 2013.0). All standard curves, generated by linear regression analysis of the plotted points, yielded strong linearity with correlation coefficients (R^2^) of ≥0.998 (Supplementary Fig. 1). Combined with amplification efficiencies over 0.90, these parameters qualify the standards for reliable gene copy number quantification (Svec et al. 2015).

#### Absolute quantification of gene copy number in qPCR

To determine the optimal sample concentration, providing a Cq value falling within the standard curve range for each gene, DNA pools of isolates Hv178a and Hv178 were prepared by combining 1 ng/µL from 2 biological replicates per isolate, followed by serial dilution to obtain the following concentrations: 1, 0.1, 0.01, and 0.001 ng/µL. Each DNA pool was run in triplicate with the primers E33, BC6, and B17.1 as previously described. The amplification efficiency for each gene was determined using LinRegPCR (v2013.0). Henceforth, qPCR runs of 4 biological replicate samples from isolates Hv178a and Hv178 at 0.1 ng/µL, with 3 technical replicates for each sample, were performed with the primers E33, BC6, and B17.1 and analyzed as previously described. Dissociation curves (Supplementary Fig. 2) and agarose gel electrophoresis were used to assess the presence of nonspecific PCR products. Mean Cq values from replicates (standard deviation ≤0.5) were used to calculate the copy number of each gene based on the corresponding standard curves. For each isolate, 3 biological replicates were selected for subsequent calculations. Gene copy number ratios were calculated between Hv178a and Hv178 for each target gene. To normalize gene copy number values considering the genome size of the 2 isolates, 2 alternative approaches were employed to estimate the number of genome copies present in the DNA template. In the first approach, genome copy numbers were determined by relating the amount of DNA template to the genome size of each isolate, as described by the following formula:


$$Genome\;copies=\;\frac{(ng\;of\;DNA)\times (6.022\times 10^{23)}}{(genome\;size\;in\;bp)\times (1\times 10^{9})\times 660}$$

Genome sizes (Hv178a–772 Mbp; Hv178–774 Mbp) previously determined by flow cytometry were used (Laureano 2024). In the second approach, calculations were made using the mass (ng) equivalent to the 2 haploid nuclei of each isolate (Doležel et al. 2007), as follows:


$$Genome\;copies=\;\frac{\left(ng\;of\;DNA\right)}{\left(2\;haploid\;nuclei\;in\;ng\right)}$$

The calculation of the mass of the 2 haploid nuclei was based on the size of each isolate's genome and the value of 1C (picograms of DNA in a haploid nucleus), using the conversion factor 1pg = 978 Mbp (Doležel and Bartoš 2005). Absolute copy number was then divided by the genome copies retrieved from each formula, and the copy number ratios between Hv178a and Hv178 were calculated.

## Results and discussion

### A complete pseudo-phased genome for *Hemileia vastatrix* (CIFC Hv178a)

We have assembled and pseudo-phased the dikaryotic genome for *Hemileia vastatrix* (CIFC Hv178a). The assembly used 200X coverage PacBio Sequel continuous long reads followed by extensive curation and then scaffolding chromosomes with chromatin conformation reads. Our genomes assembled to ∼660 Mbp, smaller than previously determined with flow cytometry at 796 Mb (Tavares et al. 2014). The most recently published *H. vastatrix* genome appears to confirm the 1C size at 775 Mbp (Ángel C. et al. 2023); however, this genome is presented as a collapsed haploid assembly. It is likely that our smaller genome size is due to the earlier-generation sequence technology data, known to be more error-prone, and possible collapsing of highly repetitive regions during the assembly, polishing, and scaffolding process. Indeed, our analysis predicted a genome size of 751.6 Mbp (hap A) to 767.2 Mbp (hap B), with an estimated empirical single-copy sequencing depth of 157 X (data not shown), though our final assemblies are smaller. Conversely, it is also possible that the genome size reported by Ángel C. et al. (2023) is inflated due to the inclusion of haplotype-variable contigs within the collapsed assembly. Despite the size discrepancy, our Hv178a genomes show good statistical completeness with BUSCO (Simão et al. 2015; Manni et al. 2021; Tegenfeldt et al. 2025) results of 80.5% and 76% using the genome mode (Table 2). Lower statistics using the protein mode (Table 2) may indicate evolutionary differences in predicted conserved single-copy genes within *H. vastatrix* compared to those in the basidiomycota database, as described in the limitations for BUSCO analyses (Manni et al. 2021).

**Table 2.** *Hemileia vastatrix* (strain CIFC Hv178a) HiC scaffolded pseudo-phased genome assembly statistics using Quast (Gurevich et al. 2013) (Gurevich et al. 2013) and BUSCO v 6.0 (basidiomycete_odb12) (Tegenfeldt et al. 2025), and annotation with Funannotate (Palmer and Stajich 2023) and EffectorP (Sperschneider and Dodds 2022).

Genome	Total length	Scaffolds	Largest scaffold	N50	L50	GC (%)
Haplotype A	665.9 Mb	76	70 Mb	35.3 Mb	8	33.5
Haplotype B	638.7 Mb	31	62 Mb	34.7 Mb	8	33.5
BUSCO	Complete	Single	Duplicated	Fragmented	Missing	Total (*n*)
Haplotype A (genome)	80.5%	78.0%	2.4%	2.9%	16.6%	2409
Haplotype B (genome)	76.0%	73.7%	2.3%	3.1%	20.9%	2409
Haplotype A (protein)	60.0%	58.8%	1.2%	10.3%	29.7%	2409
Haplotype B (protein)	60.7%	59.3%	1.5%	7.8%	31.5%	2409

Annotation	Predicted proteins	Predicted apoplastic effectors		Predicted cytoplastic effectors		Predicted total effectors
Haplotype A	13,760	101		351		452
Haplotype B	17,998	90		406		496

Repeats	LTR *Gypsy*-type repeats	LTR *Copia*-type repeats	Unknown repeats	Simple repeats	Other repeats	Total Repeats
Haplotype A	53.2%	2.7%	36.7%	0.9%	0.7%	94.2%
Haplotype B	53.5%	2.6%	36.6%	0.9%	0.7%	94.3%

Mb, megabase pairs.

### *Hemileia vastatrix* has 18 haploid chromosomes

The final Hv178a assembly has 18 haploid pseudochromosomes, clearly represented in the heatmaps (Fig. 2), a number that is consistent with other rust fungi within the Pucciniomycotina (Schwessinger et al. 2020; Duan et al. 2022; Edwards et al. 2022). Until now, chromosome numbers have not been clearly assigned to this evolutionary group of rust-forming basidiomycetes. An earlier cytological study reported the difficulty in determining a conclusive number of chromosomes for *H. vastatrix* and suggested as the best-resolved data the presence of 14 chromosomes (Chinnappa and Sreenivasan 1965). More recently, Tavares et al. (2013) determined between 7 and 13 chromosomes using both electrophoretic and cytological karyotyping, as well as fluorescence *in situ* hybridization of 2 races of *H. vastatrix* (race II—isolate 1,065 and race VI—isolate 71) from the CIFC/ISA collection. However, our results show that, despite being an ancestral rust from the family Mikronegeriaceae, chromosome numbers appear to be highly conserved. Genome data for Hv race I (Ángel C. et al. 2023) produced 11 telomere-to-telomere and 7 near-complete chromosomes, suggesting that further curation would align with the numbers that we determined for Hv178a.

**Fig. 2.**
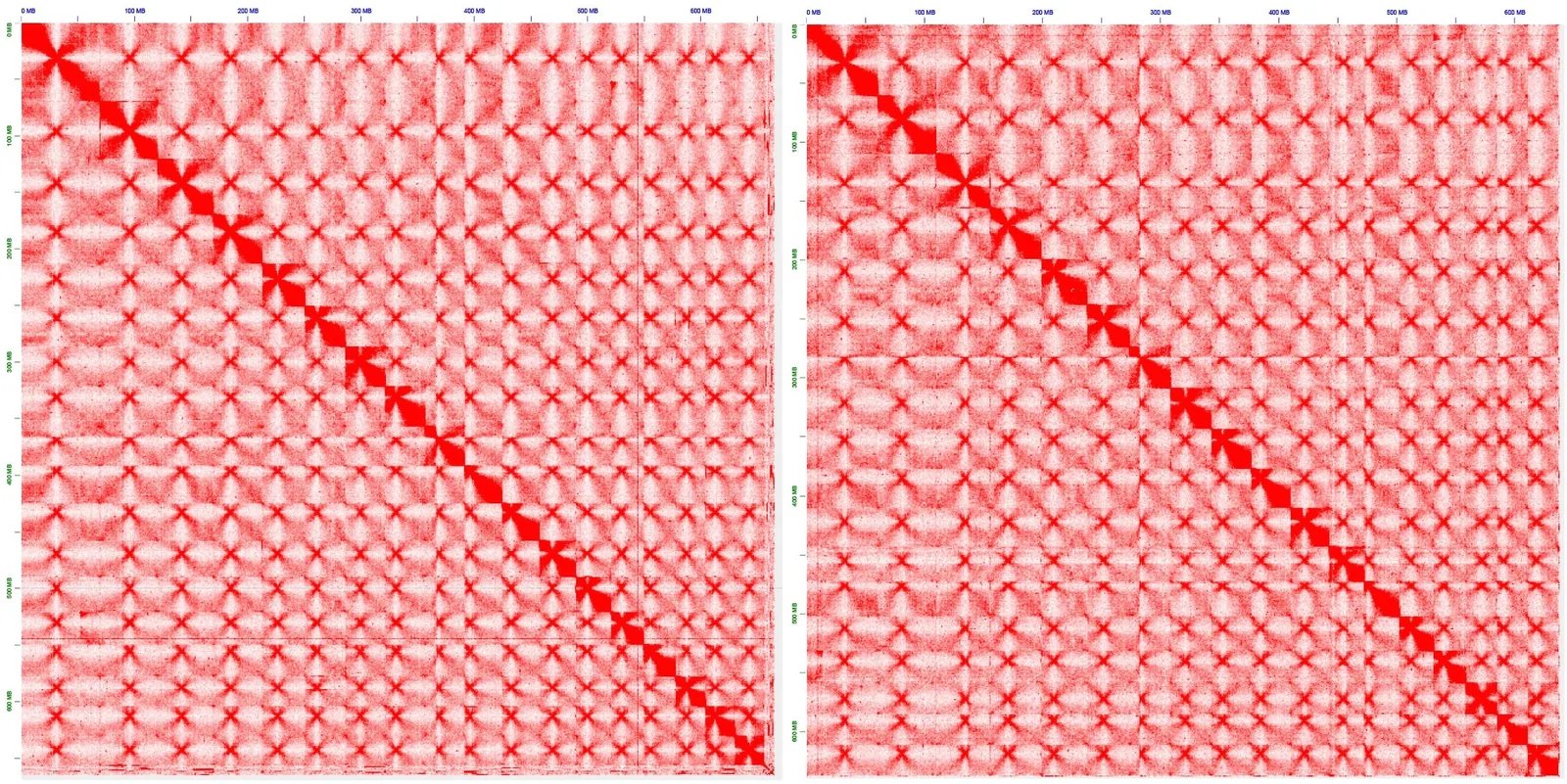
Heatmap image from juicebox visualization software (v 1.11.08) of the scaffolded *Hemileia vastatrix* (strain CIFC Hv178a) genome assembly shows 18 chromosomes visible in pseudo-haplotype A (left) and B (right).

### Repeat annotation reveals extensive *Gypsy*-type retrotransposons

The Hv178a di-haploid genomes were found to be extremely repetitive at 94.2% and 94.3% for haplotype A and B, respectively, with low guanine-cytosine (GC) content at 33.5%. Similarly, Ángel C. et al (2023) determined 81.7% masked repeat sequences within Hv race I. On investigation of the class of repeat elements, we determined 196,041 (354,456,820 bp) and 188,931 (341,578,167 bp) *Gypsy*-type long terminal repeat retrotransposons (LTR) as well as 15,336 (17,922,787 bp) and 16,350 (16,584,196 bp) *Copia-*type LTRs. In all, we determined ∼56% LTR retrotransposons and ∼34% unclassified repeats for each haplotype. Based on Cristancho et al. (2014) and Porto et al. (2019) genome assemblies, Orozco-Arias et al.(2022) improved the annotation of transposable elements (TEs) in *H. vastatrix* showing that lineages from the Gypsy superfamily correspond to 93.3% of all the LTR-RTs identified. The expansion of TE classified within the *H. vastatrix* genome is comparable to other large rust fungal genomes such as *Austropuccinia psidii* (Tobias et al. 2021), *Phakopsora pachyrhizi* (Gupta et al. 2023), and *Puccinia polysora* (Liang et al. 2023). According to Orozco-Arias et al. (2022), the recent activity of 3 Gypsy lineages (CO-HUI, Soroa, and V_Clade) might be at the origin of the genome size expansion of *H. vastatrix*. Expansion of transposable elements has been suggested to play a role in host range adaptation (Gupta et al. 2023), and the low GC content may be a result of DNA methylation silencing of TEs leading to deamination of methylated cytosines over time, as proposed for *A. psidii* (Tobias et al. 2021). In *P. pachyrhizi* (Gupta et al. 2023), the patterns of expression of TEs were studied during the infection cycle and a subset determined to be highly expressed *in planta* at 24 h post inoculation. It would be interesting to determine if a similar pattern of gene expression is observed during infection on *C. arabica*, particularly on differential host plants, as a potential explanation for new virulence mechanisms.

### Pseudo-haplotype orthologs show no TE enrichment in singletons

We tested unassigned singletons from our ortholog analysis for TE enrichment to investigate adaptive genomic potential (Torres et al. 2021). Singletons represent a gene for which there is exactly 1 copy in the target organism's genome and no detectable homologs in the rest of the dataset (Sun et al. 2023). We tested the 4,099 and 5,383 singleton proteins from pseudo-haplotype A and B, respectively. We found no statistical significance of TE enrichment using Fisher's Exact Test in either haplotype (TE Enrichment for Haplotype A: *P*-value = 0.37, and Haplotype B: *P*-value = 0.89). Ortholog collinearity visualization (Sun et al. 2023) indicated syntenic and non-syntenic blocks within and between chromosomes, indicating likely unresolved assembly and phasing errors. An example of phase-block crossover between chromosome 1 and 17 is represented in the Supplementary Data (Supplementary Fig. 3) and for all other chromosomes here: https://osf.io/wtn4h/files/osfstorage.

### Predicted protein-coding gene numbers are different in each pseudo-haplotype

We annotated the protein-coding genes within each pseudo-haplotype and predicted 13,760 and 17,998 in haplotype A and B, respectively. The discrepancy may be due to assembly or annotation errors, or they may have a biological explanation. Haploid nuclei of dikaryotic rust fungi, where the 2 haploid nuclei coexist within the same cell, can evolve semi-independently over long evolutionary periods. High-quality phased genome assemblies of rust fungi were able to demonstrate that the 2 haplotypes may display substantial sequence divergence as well as structural variation, gene presence–absence polymorphisms, and haplotype-specific genes (Wang et al. 2024; Luo et al. 2025). The difference in numbers of annotated genes between pseudo-haplotypes in *H. vastatrix* could be difficult to understand given the lack of any known sexual stage, albeit population genomic footprints of hybridization and recombination were previously detected (Silva et al. 2018). However, long-term asexual reproduction can lead to divergent evolution within the 2 dikaryotic nuclei by promoting a continuous accumulation of mutations and TEs, structural variations, and a high level of heterozygosity, as shown in asexual lineages of *P. striiformis* f.sp*. tritici* isolates (Schwessinger et al. 2020). While these explanations can be considered, our current genomes are not fully phased and therefore haploid-specific results are not definitive.

We processed the amino acid sequences to predict subcellular localization based on known signal peptides (Nielsen 2017). Based on the outputs of mature proteins that had no recognizable transmembrane signal peptide, we predicted 452 and 496 virulence molecules, known as effectors, in pseudo-haplotypes A and B. Effectors are characterized as secreted proteins that facilitate infection and suppress host defenses (Sperschneider and Dodds 2022). The predicted effectors were classified as either cytoplasmic or apoplastic based on machine learning approaches. This method identified 351 (406) cytoplasmic and 101 (90) apoplastic effectors in pseudo-haplotypes A and B. Based on 15 obligate biotroph genomes tested, average percentages of cytoplasmic and apoplastic predicted effectors are 32.5% and 11.3%, respectively (Sperschneider and Dodds 2022). In the haploid genomes for Hv178a, the percentages of cytoplasmic and apoplastic predicted effectors are 44.0% and 12.7% (haplotype A), 45.6% and 10.1% (haplotype B), which is more comparable to the finding of 40% apoplastic effectors determined for *A. psidii*, a similarly larger genome for an obligate biotroph (Tobias et al. 2021). Using ChromoMap (Anand and Rodriguez Lopez 2022), we show the predicted effector locations and numbers are different across chromosome pairs, indicating that the 2 nuclei likely do not interact and are on their own evolutionary path (Fig. 3). The differences observed here suggest that the nuclei of *H. vastatrix* may harbor partially distinct virulence repertoires, potentially increasing the adaptive potential of this obligate biotrophic pathogen.

**Fig. 3.**
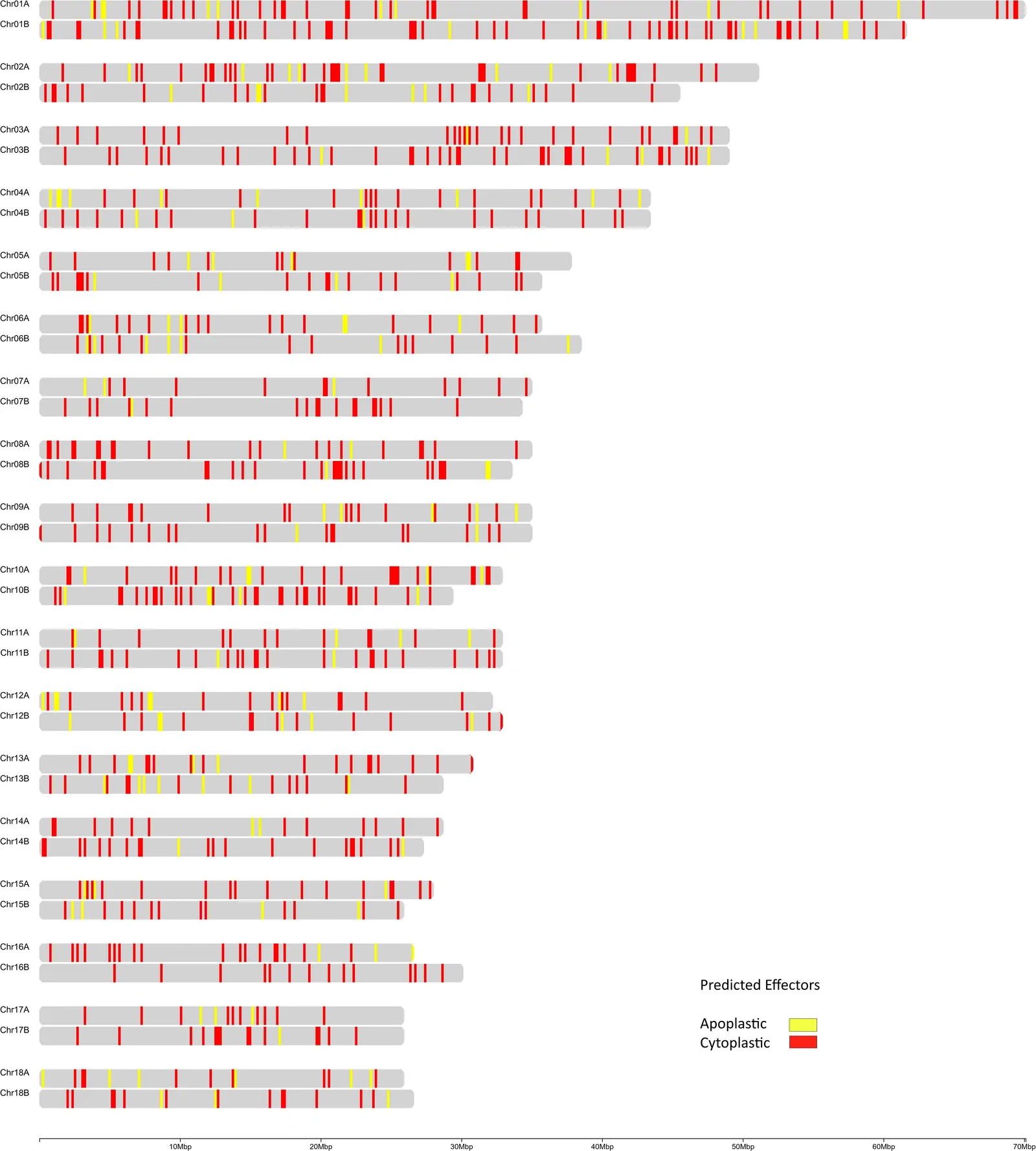
Predicted cytoplasmic (red) and apoplastic (yellow) effector gene locations on the pseudo-phased chromosome pairs for CIFC *Hemilieia vastatrix* 178a visualized using ChromoMap (Anand and Rodriguez Lopez 2022). Lines represent single predicted genes.

### Read depth analysis suggests chromosome 17 trisomy in Hv178a

To investigate whether any genes had gained or lost copy number in Hv178a, the CNR of Hv178a compared to 11 other *H. vastatrix* isolates (Hv23, Hv70, Hv71, Hv178, Hv264a, Hv741, Hv995, Hv999, Hv1427, Hv3305, and HvI) was calculated for each predicted gene (Supplementary Data 1, BUSCO_table and Gene_Table). To identify potential regions of interest, log2 CNR was plotted against chromosomal position for each chromosome (Supplementary Fig. 4). For most chromosomes, such as chromosome 1, log2 CNR was around 0 across the length of the chromosome (Fig. 4a). However, chr17 displayed a markedly different pattern, with a consistent elevation of 0.5 or more (Fig. 4b). This corresponded to a CNR value of 1.4 to 1.5. Given the noisiness of the CN estimates, which were based on short-read data rather than more consistently sequenced long reads, this was highly suggestive of a 50% increase across the entire chromosome, which could be explained by a third copy (trisomy) of chr17 in the genome. Despite an elevated fold change for genes along the entire chr17, no clear clusters of predicted effectors were observed when plotted on the chromosome.

**Fig. 4.**
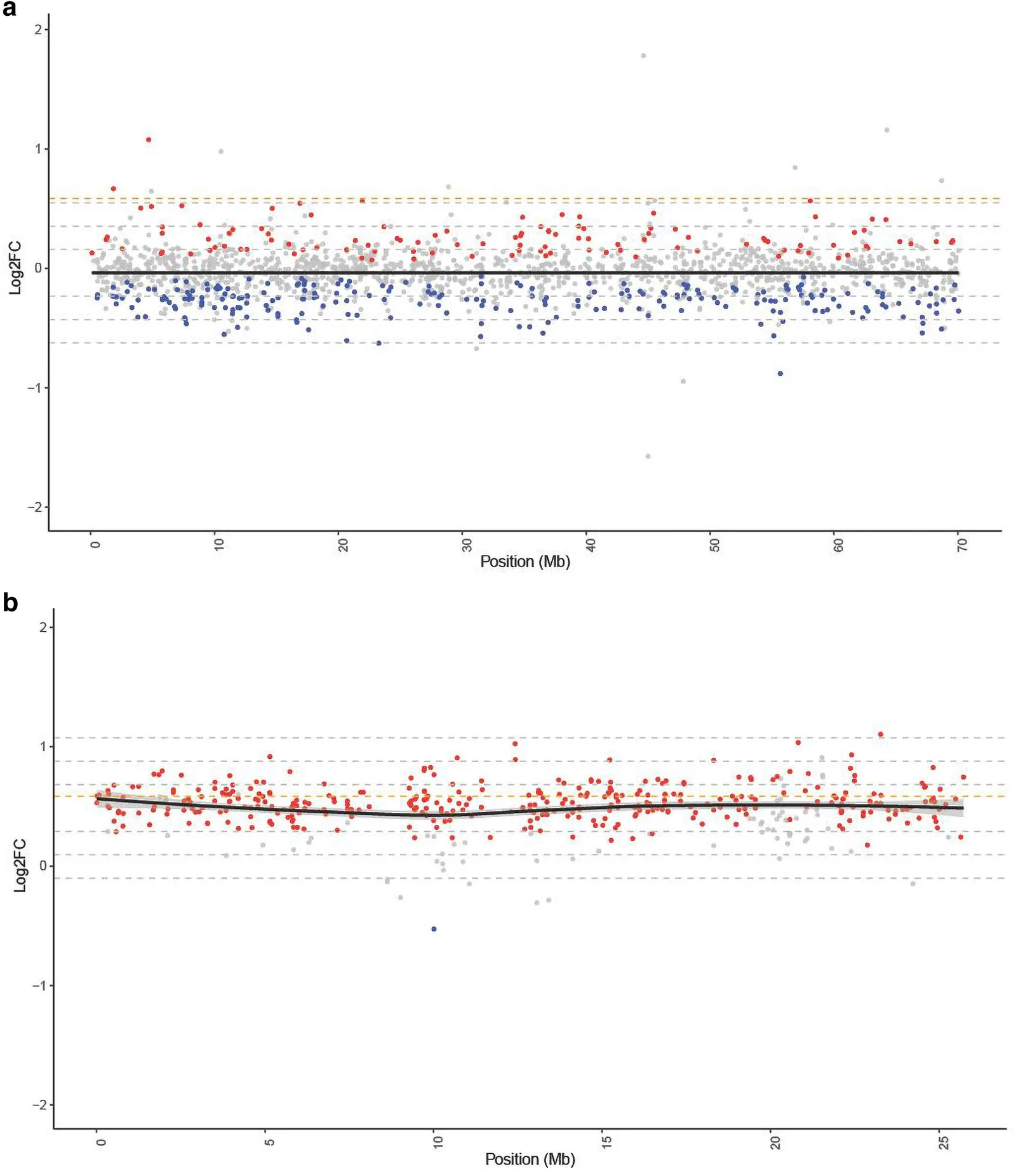
Hv178a Log2 copy number ratio compared to the mean of 11 other Hv strains. a. Chromosome 1, b. Chr17. The CN ratio was calculated as the CN for Hv178a vs the mean of 11 other *H. vastatrix* isolates: Hv23, Hv70, Hv71, Hv178, Hv264a, Hv741, Hv995, Hv999, Hv1427, Hv3305 and HvI. A log2 CN ratio of 0 indicates no CN difference. Each dot is a gene, plotted against chromosomal position. Red: Hv178a has the highest CN of all strains; Blue: Hv178a had the lowest CN of all strains; Gray: Hv178a is not the most extreme CN. Black line: loess regression line of log2 CNR; Gray lines, 1 to 3 standard deviations from the mean. For all chromosome plots, see Supplementary Fig. 4.

### Significant protein domain CN fold change in Hv178a compared to other isolates

Fold change analysis of Pfam domain families, between the mean of Hv178a and 11 other isolates, showed domains both increasing and decreasing in copy number. The most extreme increase was for 8 domain families that had a Log2 fold change between 8 and 9. The top 10 examples of CN fold change in Hv178a protein domains indicated a significant increase in Cytochrome C subunits when compared to other isolates (Table 3).

**Table 3.** Top 10 fold changes observed for mean copy number of genes grouped by protein domains.

	Pfam name	Database ID	Domains	Mean CN Hv178a	Mean CN 11 isolates	Log2 fold change	*P*-value
	Cytochrome C oxidase subunit II, periplasmic domain	PF00116	1	1.95	0.00	8.74	1.00E−36
**Top 10 fold change incr.**	NADH dehydrogenase	PF00146	1	1.95	0.00	8.74	1.00E−36
	Cytochrome C oxidase subunit III	PF00510	2	5.13	0.02	8.37	1.00E−36
	Cytochrome b(C-terminal)/b6/petD	PF00032	2	4.93	0.02	8.32	1.00E−36
	LAGLIDADG endonuclease	PF00961	3	5.92	0.02	8.30	1.00E−36
	Cytochrome C and quinol oxidase polypeptide I	PF00115	2	5.26	0.02	8.18	1.00E−36
	Proton-conducting membrane transporter	PF00361	4	4.96	0.02	8.15	1.00E−36
	LAGLIDADG DNA endonuclease family	PF03161	4	5.02	0.02	8.06	1.00E−36
	Stage II sporulation protein E (SpoIIE)	PF07228	1	1.21	0.47	1.36	0.004227
	PPP5 TPR repeat region	PF08321	1	1.15	0.46	1.34	0.007755
	DASH complex subunit Dad2	PF08654	1	0.11	0.99	−3.17	0.000435
**Top 10 fold change decr.**	PAC2 family	PF09754	1	0.12	1.00	−3.06	3.62E−10
	Nuclear cap-binding protein subunit 3	PF10309	1	0.17	1.35	−2.99	0.012596
	YEATS family	PF03366	1	0.21	0.97	−2.20	1.59E−05
	Small subunit of serine palmitoyltransferase-like	PF11779	1	0.32	1.10	−1.78	0.010667
	Tho complex subunit 7	PF05615	1	0.34	1.15	−1.76	3.49E−10
	Helix-turn-helix	PF01381	1	0.34	1.11	−1.70	0.001031
	Multiprotein bridging factor 1	PF08523	1	0.34	1.11	−1.70	0.001031
	GTP-binding protein TrmE N-terminus	PF10396	1	0.36	1.15	−1.68	1.57E−07
	MnmE helical domain	PF12631	1	0.36	1.15	−1.68	1.57E−07

Incr., increase; decr., decrease.

### qPCR data support Chr17 trisomy in isolate Hv178a

The putative trisomy of chr17 in isolate Hv178a was further evaluated by qPCR absolute quantification of target gene copy number, using isolate Hv178—the original field-collected strain from which the mutant Hv178a was produced- as the reference. Under the hypothesis of chr17 trisomy, loci located on chr17 are expected to be present in 3 copies in the trisomic isolate vs 2 copies in the euploid isolate, while loci on other chromosomes are expected to remain disomic in both isolates. Accordingly, when normalizing absolute copy numbers between isolates, a ratio of approximately 1.5 (3:2) should be observed for both chr17 loci analyzed (effector gene E33 and BUSCO gene B17.1), whereas a ratio of approximately 1.0 (2:2) should be observed for the chromosome 6 BUSCO gene (BC6), serving as a disomic reference chromosome. Indeed, estimation of absolute gene copy number for these 3 gene targets showed ratios between Hv178a and Hv178 of 1.601 ± 0.311for E33, 1.220 ± 0.311 for BC6 and 1.472 ± 0.384 for B17.1. These proportions are maintained with very similar values when a genome size normalization factor is additionally applied (Table 2). Although a previous estimate of the genome size for Hv178a was reported by Tavares et al. (2014) as 796.8 Mbp, we used the values reported by Laureano (2024) for normalization purposes: 772 Mbp for Hv178a and 774 Mbp for Hv178. These estimates were selected because they were based on a larger number of replicates for Hv178a (*n* = 10 vs *n* = 5 in Tavares et al. 2014) and because they provide the only available genome size estimate for Hv178. Nevertheless, we also calculated the absolute copy number normalized using the genome size reported by Tavares et al. (2014), and no significant differences in ratios were observed (E33, 1.648 ± 0.320; BC6, 1.256 ± 0.331; B17.1, 1.515 ± 0.395). This consistent observation of a ∼1.5-fold increase in absolute copy number for chr17 loci, coupled with a ∼1.0 ratio for the chromosome 6 control locus, provides quantitative evidence supporting trisomy of chr17 in Hv178a (Supplementary Table 1). This dual-locus strategy on the same chromosome, combined with an independent chromosomal control, minimizes locus-specific amplification bias and strengthens the inference of chromosomal copy number variation.

Collectively, these findings are consistent with a third copy of chr17 in Hv178a, which could mean that the acquisition of increased virulence by an additional virulence factor (v4) in relation to Hv178 was accompanied by a catastrophic genomic event. Such extensive rearrangements may be more common in coffee rust than previously anticipated, as other mutant isolates derived from adaptation experiments made at CIFC exhibit substantial genome size differences; for example, Hv264 (v_1,4_) to Hv264a (v_1,4,6_) show an increase of 36 Mbp (Laureano 2024). These results suggest that host adaptation may act as a catalyst for large-scale genomic restructuring. The extra chr17 in Hv178a may similarly expand the genomic landscape available for effector gene diversification and expression, analogous to how mini-chromosomes in *M. oryzae* function as fast-evolving genomic compartments driving host adaptation (Ma and Xu, 2019; Peng et al. 2019). Genomic regions with elevated structural plasticity are hotspots for generating virulence-associated variation, enabling rapid responses to host immune pressure. Such change could potentially increase the copy number, dosage imbalance, and combinatorial function of effector loci, enhancing the pathogen’s ability to evade host recognition or suppress immunity, contributing to increased virulence.

## Conclusion

We have assembled a chromosome-level, pseudo-phased and annotated genome for *Hemileia vastatrix* isolate Hv178a. Our research determined that the genome is distributed across 18 haploid chromosomes, in line with other rust fungal pathogens. An interesting finding of chr17 haploid trisomy for Hv178a, not present in the ancestral Hv178 strain, suggests a potential explanation for the changed virulence profile in this isolate.

## Supplementary Material

jkag214_Supplementary_Data

## Data Availability

Raw data are available at the following BioProject accession number at the National Center for Biotechnology Information (NCBI): PRJNA837996. The genomes have been deposited under the accessions PRJNA851216, JAMZDC000000000, and PRJNA851215, JAMZDD000000000. Protein annotation files, including predicted effector data, are available at: https://osf.io/wtn4h/files/osfstorage. Isolate data used for annotation and for copy number analysis are available at the following NCBI accession numbers: PRJNA1483988 and PRJNA1484073. Supplemental material available at G3 online.
