# Supplementary material for: A pseudo-phased genome assembly for Hemileia vastatrix reveals an isolate-specific chromosomal haploid trisomy
Source: G3 (Bethesda). 2026 Aug 1;16(10):jkag214. doi: 10.1093/g3journal/jkag214 (PMC13643790; doi:10.1093/g3journal/jkag214)
Supplement: Supplementary file 1 [file jkag214_supplementary_data.zip › Supplementary_Figure_1_G3-2026-407076.docx]

Supplementary Figure 1. Standard curves of the plasmid copy numbers for each target gene obtained by plotting the average Cq of 6 points against the logarithm of the number of copies in 1 µL for each sample: E33 (A); BC6 (B); B17.1 (C). Each curve presents its own equation and R^2^.
