# Supplementary material for: A pseudo-phased genome assembly for Hemileia vastatrix reveals an isolate-specific chromosomal haploid trisomy
Source: G3 (Bethesda). 2026 Aug 1;16(10):jkag214. doi: 10.1093/g3journal/jkag214 (PMC13643790; doi:10.1093/g3journal/jkag214)
Supplement: Supplementary file 1 [file jkag214_supplementary_data.zip › Supplementary_Figure_2_G3-2026-407076.docx]

Supplementary Figure 2. Amplification curves and dissociation curves from qPCR analysis of Hv178a and Hv178 samples at 0.1 ng/µL for E33 (A), BC6 (B), and B17.1 (C) target genes. Samples of Hv178a are shown in blue, while samples of Hv178 are shown in orange. The negative control is shown in yellow. (Bio-Rad CFX Maestro 1.0 software, version 4.0.2325.0418, Bio-Rad, USA).
