# Supplementary material for: A pseudo-phased genome assembly for Hemileia vastatrix reveals an isolate-specific chromosomal haploid trisomy
Source: G3 (Bethesda). 2026 Aug 1;16(10):jkag214. doi: 10.1093/g3journal/jkag214 (PMC13643790; doi:10.1093/g3journal/jkag214)
Supplement: Supplementary file 1 [file jkag214_supplementary_data.zip › Supplementary_Material_Legends_G3-2026-407076.docx]

### **Supplementary Material Legends**

Supplementary Figure 1. Standard curves of the plasmid copy numbers for each target gene obtained by plotting the average Cq of 6 points against the logarithm of the number of copies in 1 µL for each sample: E33 (A); BC6 (B); B17.1 (C). Each curve presents its own equation and R2.

Supplementary Figure 2. Amplification curves and dissociation curves from qPCR analysis of Hv178a and Hv178 samples at 0.1 ng/µL for E33 (A), BC6 (B), and B17.1 (C) target genes. Samples of Hv178a are shown in blue, while samples of Hv178 are shown in orange. The negative control is shown in yellow. (Bio-Rad CFX Maestro 1.0 software, version 4.0.2325.0418, Bio-Rad, USA).

Supplementary Figure 3. Ortholog collinearity visualisation with chromosome-level scaffolds in OrthoVenn3 (Sun et al., 2023) shows synteny blocks within and between chromosomes 1 and 17. Visualisation of all chromosomes share this pattern indicating some unresolved assembly and phasing of the haploid genomes.

Supplementary Figure 4. Chromosome Plots of Fold Change of Hv178a vs Mean other strains using mapped short read data (SR). Chromosome 1 to 18. Red: Hv178a is the highest Copy Number when compared to other strains, Blue: Lowest Copy Number when compared to other strains, Grey: Hv178a is not the most extreme when compared to other strains. Black Regression Line formed by geom_smooth (loess), grey lines indicate Standard Deviations 1, 2 ,3 from the mean. Orange line indicates 50% increase in Copy Number.

Supplementary Table 1. Estimation of absolute copy number in isolates Hv178 and Hv178a, and derived ratios, for the gene targets studied using three different approaches: direct calculation from the standard curves (Absolute copies), normalization by the respective genome size of each isolate (Copies/genome size/ng), and normalization by the respective genome mass of each isolate based on the value of 1C in pg (Copies/genome mass/ng).

Supplementary Data 1. BUSCO_Table - copy number data and statistics of genes (BUSCOs) for isolates compared to Hv178a haplotype A. The Copy Number Ratio was compared to eleven other H. vastatrix isolates (Hv23, Hv70, Hv71, Hv178, Hv264a, Hv741, Hv995, Hv999, Hv1427, Hv3305 and HvI) and calculated for each gene. Gene_Table - copy number data and statistics of annotated genes and predicted effectors for isolates compared to Hv178a haplotype A. The Copy Number Ratio was compared to eleven other H. vastatrix isolates (Hv23, Hv70, Hv71, Hv178, Hv264a, Hv741, Hv995, Hv999, Hv1427, Hv3305 and HvI) and calculated for each gene.
